# Natural Teeth Present and Quality of Life in Older Adults from Indigenous Communities in the Peruvian Amazon: A Cross-Sectional Study

**DOI:** 10.3290/j.ohpd.c_2833

**Published:** 2026-09-29

**Authors:** Yshoner Antonio Silva-Diaz, Cintya Elisabeth Odar-Rojas, Ruth Clarivel Vega-Rojas, José Luis Rodriguez Medina, Lenin Edwads Velez Rodríguez, Clelia Jima Chamiquit

**Affiliations:** a Yshoner Antonio Silva-Diaz Professor, Grupo de Investigación Plantas Medicinales y Medicina Alternativa (PYMA), Centro de Investigación Plantas Medicinales, Terapias Alternativas y Comunidades Nativas y Rurales (CIPMAYCOM), Instituto de Salud Integral Intercultural (ISI), Facultad de Ciencias de la Salud (FACISA), Universidad Nacional Toribio Rodríguez de Mendoza (UNTRM), Amazonas, Perú. [Research Group on Medicinal Plants and Alternative Medicine (PYMA), Research Center for Medicinal Plants, Alternative Therapies, and Native and Rural Communities (CIPMAYCOM), Institute of Comprehensive Intercultural Health (ISI), Faculty of Health Sciences (FACISA), National University Toribio Rodríguez de Mendoza (UNTRM), Amazonas, Perú] Conceptualization, methodology, wrote original draft.; b Cintya Elisabeth Odar-Rojas Lecturer, Grupo de Investigación Plantas Medicinales y Medicina Alternativa (PYMA), Centro de Investigación Plantas Medicinales, Terapias Alternativas y Comunidades Nativas y Rurales (CIPMAYCOM), Instituto de Salud Integral Intercultural (ISI), Facultad de Ciencias de la Salud (FACISA), Universidad Nacional Toribio Rodríguez de Mendoza (UNTRM), Amazonas, Perú; Escuela Profesional de Psicología, Facultad de Ciencias de la Salud (FACISA), Universidad Nacional Toribio Rodríguez de Mendoza (UNTRM), Amazonas, Perú. [Research Group on Medicinal Plants and Alternative Medicine (PYMA), Research Center for Medicinal Plants, Alternative Therapies, and Native and Rural Communities (CIPMAYCOM), Institute of Comprehensive Intercultural Health (ISI), Faculty of Health Sciences (FACISA), National University Toribio Rodríguez de Mendoza (UNTRM), Amazonas, Perú; Professional School of Psychology, Faculty of Health Sciences (FACISA), National University Toribio Rodríguez de Mendoza (UNTRM), Amazonas, Perú] Conceptualization, visualization, reviewed and edited the manuscript.; c Ruth Clarivel Vega-Rojas Lecturer, Grupo de Investigación Plantas Medicinales y Medicina Alternativa (PYMA), Centro de Investigación Plantas Medicinales, Terapias Alternativas y Comunidades Nativas y Rurales (CIPMAYCOM), Instituto de Salud Integral Intercultural (ISI), Facultad de Ciencias de la Salud (FACISA), Universidad Nacional Toribio Rodríguez de Mendoza (UNTRM), Amazonas, Perú. [Research Group on Medicinal Plants and Alternative Medicine (PYMA), Research Center for Medicinal Plants, Alternative Therapies, and Native and Rural Communities (CIPMAYCOM), Institute of Comprehensive Intercultural Health (ISI), Faculty of Health Sciences (FACISA), National University Toribio Rodríguez de Mendoza (UNTRM), Amazonas, Perú] Wrote original draft, validation, reviewed and edited the manuscript.; d José Luis Rodriguez Medina Professor, Facultad de Ciencias Económicas y Administrativas, Universidad Nacional Toribio Rodríguez de Mendoza (UNTRM), Amazonas, Perú. [Faculty of Economic and Administrative Sciences, National University Toribio Rodríguez de Mendoza (UNTRM), Amazonas, Perú.] Conceptualization, methodology, and visualization.; e Lenin Edwads Velez Rodríguez Dentist, Grupo de Investigación Plantas Medicinales y Medicina Alternativa (PYMA), Centro de Investigación Plantas Medicinales, Terapias Alternativas y Comunidades Nativas y Rurales (CIPMAYCOM), Instituto de Salud Integral Intercultural (ISI), Facultad de Ciencias de la Salud (FACISA), Universidad Nacional Toribio Rodríguez de Mendoza (UNTRM), Amazonas, Perú; Escuela Profesional de Estomatología, Facultad de Ciencias de la Salud (FACISA), Universidad Nacional Toribio Rodríguez de Mendoza (UNTRM), Amazonas, Perú. [Research Group on Medicinal Plants and Alternative Medicine (PYMA), Research Center for Medicinal Plants, Alternative Therapies, and Native and Rural Communities (CIPMAYCOM), Institute of Comprehensive Intercultural Health (ISI), Faculty of Health Sciences (FACISA), National University Toribio Rodríguez de Mendoza (UNTRM), Amazonas, Perú. Professional School of Stomatology, Faculty of Health Sciences (FACISA), National University Toribio Rodríguez de Mendoza (UNTRM), Amazonas, Perú] Validation, formal analysis, and investigation.; f Clelia Jima Chamiquit Professor, Facultad de Ciencias de la Salud (FACISA), Universidad Nacional Toribio Rodríguez de Mendoza (UNTRM), Amazonas, Perú [Faculty of Health Sciences (FACISA), National University Toribio Rodríguez de Mendoza (UNTRM), Amazonas, Perú.] Validation, wrote original draft.

**Keywords:** aged, edentulous, indicators of quality of life, indigenous peoples, quality of life.

## Abstract

**Purpose:**

To analyze the association between the number of natural teeth and quality of life, and to identify the prevalence of edentulism among older adults in two indigenous communities in the Peruvian Amazon.

**Materials and Methods:**

This was a cross-sectional observational study conducted in adults over 60 years of age from the communities of Imacita and Yamayakat. Quality of life was assessed using the WHOQOL-BREF questionnaire adapted for indigenous communities, and the number of natural teeth present was recorded using a dental chart.

**Results:**

A total of 54 participants from the Imacita community and 41 from Yamayakat were included. The prevalence of edentulism was higher in Yamayakat, n=18 (43.90%), compared to Imacita, n=14 (25.93%). The number of natural teeth present was significantly associated with satisfaction with general health (β = 1.85; 95% CI: 1.37–2.34; p<0.001), perceived overall quality of life (β = 1.54; 95% CI: 1.14–1.95; p<0.001), physical domain (β = 0.81; 95% CI: 0.51–1.10; p<0.001), and social domain (β = 0.72; 95% CI: 0.26–1.18; p=0.002).

**Conclusions:**

An association was observed between the number of teeth present and the quality of life of older adults, while the prevalence of edentulism did not show significant differences between the two communities. These findings highlight the need to implement interventions aimed at preventing tooth loss among indigenous older adults.

Edentulism is defined as the partial or complete loss of natural teeth. Countries in Latin America and the Caribbean show a higher prevalence of edentulism, with the most affected being Bolivia at 10.59%, Peru at 9.92%, and Brazil at 8.99%.^41^ These figures are predominantly concentrated in the aging population and show a significant association with increased mortality.^16^ The primary causes of tooth loss are dental caries and periodontitis,^6^ the latter being defined as an inflammatory disease characterized by the destruction of the periodontal ligament and alveolar bone,^13^ which, if left untreated, leads to an increased risk of tooth loss.^58^ It is estimated that approximately 79.3% of older adults worldwide have periodontitis,^51^ with non-Hispanic Black adults (14.7%), Mexican American adults (13.4%), and other Hispanic adults (7.8%) experiencing it most frequently.^18^ On the other hand, 60.7% of the global population is affected by dental caries, with its prevalence increasing to 79.6% in the Americas.^1^ Dental caries is an infectious disease that destroys the tooth through the acidification of the tooth surface, resulting in the removal of minerals from the enamel and degradation of the organic matrix, thereby increasing the likelihood of edentulism.^8,48^

Likewise, tooth loss is also the result of the interaction of various biological and socioeconomic factors.^19,46^ From a biological perspective, aging leads to physiological changes, alterations in tissue repair, and reduced salivary flow,^35,43^ the decline of which creates an environment with a greater likelihood of oral diseases and progressive tooth loss.^15^ Furthermore, among the socioeconomic factors, it is noteworthy that populations with certain cultural, linguistic, and geographic characteristics^22,55^ face difficulties in accessing preventive and dental care services, disproportionately affecting this age group,^22^ which is more vulnerable to such conditions. Within this context, understanding the consequences of edentulism is important in order to improve the quality of life of older adults.

Among older adults from native or Indigenous communities, quality of life is approached holistically, integrating their physical, psychological, environmental, and social perceptions.^20,44^ Aging is associated with a progressive decline in functional capacity,^38^ which is influenced by community accessibility and the availability of health interventions; both dimensions are perceived by older adults and may affect their physical and environmental quality of life.^30,32^ Likewise, in native communities, older adults are highly valued for their role as transmitters of ancestral knowledge,^27^ which benefits their psychological and social well-being.^21,27^ However, there are certain factors that may modify quality of life, among which oral health stands out, as it can compromise nutritional status and directly affect the physical, functional, and psychological dimensions of older adults. Currently, various instruments are available to assess quality of life,^42^ with the WHOQOL-BREF being one of the most widely used and frequently adapted for diverse Indigenous communities due to its practicality and its comprehensive assessment of multiple dimensions of quality of life,^29^ making it particularly relevant for these populations.

Previous literature on Indigenous communities in India and Brazil indicates that oral health and edentulism affect quality of life, particularly in the physical and psychological domains of older adults.^34,39,50,53^ In contrast, studies conducted in China and Chile have found that having more than 20 natural teeth is associated with better physical health, likely due to the more varied diet observed among older adults.^5,49^ However, despite the available evidence, studies examining this issue specifically in Indigenous or native communities remain limited, primarily because of the limited accessibility and the cultural characteristics of these populations, which are distinctive features of communities in the Peruvian Amazon.

Therefore, the present study aims to examine the association between the number of natural teeth and quality of life, and to determine the prevalence of edentulism among older adults in two Indigenous communities in the Peruvian Amazon. The findings may help address important knowledge gaps regarding Indigenous oral health in the Latin American context by providing empirical evidence that enables a more comprehensive understanding of the relationship between tooth loss and the functional and psychosocial well-being of this population.

## MATERIALS AND METHODS

This was a cross-sectional observational study conducted in the Indigenous communities of Imacita and Yamayakat, located in the Peruvian Amazon. The sample consisted of a total of 95 participants over 60 years of age, including 54 from Imacita (representing 58.69% of the 92 older adults estimated according to the Health Center report for this community) and 41 from Yamayakat (equivalent to 69.49% of the 59 residents within the same age group in the Yamayakat community). Furthermore, the average life expectancy in the Amazonas Department is 75 years,^32^ providing context for the age profile of our study participants.

The communities are located approximately seven hours from Chachapoyas (capital of the Department of Amazonas, Peru), and the predominant language is Awajún. Imacita has a health center and an educational institution and, due to its strategic location on the banks of the Marañón River, serves as an administrative and commercial hub that facilitates coordination among communities. In contrast, Yamayakat is located approximately three minutes from Imacita by peque peque (a small motorized boat), and due to its proximity, its inhabitants travel to Imacita to access health care services.

To reduce selection bias and increase community representativeness, two main recruitment strategies were implemented: a) older adults aged 60 years and above who sought dental care at the Imacita Health Center during the period from March to June 2025; and b) residents identified through a call organized by the APU (community leader) of the Indigenous communities.

The study was approved by the Faculty of Health Sciences at the National Toribio Rodríguez de Mendoza University and by the Research Ethics Committee of Gustavo Lanatta Luján Hospital in Amazonas under approval code REPORT No. 01-2024, issued on September 5, 2024; both institutions reviewed the protocol prior to the start of data collection. The study was designed in accordance with the Declaration of Helsinki, ensuring compliance with the principles of beneficence, non-maleficence, respect, and confidentiality by the researchers toward the participants. Prior to implementation, approval was obtained from the APU of both communities. Subsequently, the informed consent process was explained to the population in the Awajún language, and understanding was verified through confirmation questions regarding the study objectives and participants’ right to withdraw voluntarily at any time without economic or social consequences.

The following inclusion criteria were established: 1) adults aged 60 years and older; 2) residents of the indigenous communities of Imacita and Yamayakat, according to the report of the Imacita Health Center; 3) verbal ability in Awajún or Spanish; and 4) acceptance of participation in the study through signing the informed consent form. Likewise, the following exclusion criteria were established: 1) a medical diagnosis of dementia or other neurological disability that prevented understanding of the study protocol or effective communication with the participant; 2) presence of deafness or mutism without communicative capacity; and 3) refusal to participate in the study.

### Data Collection Procedure

To ensure homogeneity in the assessment, all participants were evaluated by the same trained professional, who is fluent in the Awajún language and routinely practices at the health center serving this population, which facilitated effective communication throughout the entire procedure. The same data collection protocol was applied to minimize potential bias and to respect the cultural context of the population. Prior to the clinical evaluation, the quality-of-life questionnaire was administered; in cases where the older adult was not literate, assistance was provided during the response process. The clinical evaluation was performed through visual inspection without radiographs, using a lamp, a No. 5 flat mirror, and a dental explorer. During the clinical assessment, biosafety protocols were implemented for both the healthcare professional and the participants in accordance with Peruvian health regulations, ensuring proper disinfection and sterilization of the instruments used.

### Quality of Life

Quality of life is defined as a set of conditions that determine the level of well-being and satisfaction experienced by an individual.^57^ The study used the WHOQOL-BREF questionnaire,^50^ which was adapted for vulnerable Peruvian contexts.^22^ The adapted version was translated into the Awajún language; this process included linguistic adaptation and comprehension testing among community residents. The questionnaire consists of 26 items organized into the following domains: 1) Physical: comprising seven items that assess how physical health influences quality of life; 2) Psychological: comprising six items that evaluate an individual’s emotional and cognitive state; 3) Social: comprising three items that assess social support networks and interpersonal relationships; and 4) Environmental: comprising eight items that evaluate the living environment and its influence on well-being.^26^ Scoring was performed on a 0–100 scale after summing each domain, taking into account reverse-coded items.

The adapted version reported that reliability was assessed using Cronbach’s alpha coefficient, with all domains showing values above 0.70, indicating good internal consistency.^26^ Likewise, validity was supported by the original validation processes conducted by the World Health Organization for the WHOQOL-BREF and WHOQOL-OLD questionnaires.^17,24^

### Natural Teeth Present

The total number of natural teeth present was recorded according to the following criteria: 1) restored teeth, provided that at least 50% of the clinical crown structure remained visible; and 2) residual roots without mobility, inflammation, or pain. Teeth were considered absent when the following conditions were met: 1) a fully healed edentulous space; 2) residual roots with visible mobility; and 3) residual roots with inflammation or the presence of pain. All natural teeth were included in the count, including third molars, while implants, prostheses, and bridges were excluded. The dental chart^33^ was used for recording, in which a horizontal blue line was drawn over the crowns of missing teeth. Data recording included cases with zero natural teeth present, and participants were classified into two groups: 1) completely edentulous: loss of all natural teeth; and 2) dentate: presence of at least one natural tooth or all natural teeth present.

### Data Analysis

Descriptive statistics were used, in which continuous variables were presented as means and standard deviations, while categorical variables were expressed as absolute frequencies and percentages. The normality of the data was assessed using the Shapiro–Wilk test; based on the results, Student’s t-test was used for continuous variables that followed a normal distribution, and the chi-squared test was used for categorical variables, while the Mann–Whitney U test was applied to variables that did not follow a normal distribution. To assess the association between the number of teeth present and quality of life across each domain, bivariate linear regression analysis and multivariable linear regression adjusted for age, sex, and community of residence were performed. The main variable was the number of natural teeth present, recorded using the dental chart. The tooth count was used as a continuous independent variable. In each model, the dependent variable was the score of the corresponding WHOQOL-BREF domain. The regression models were presented using unstandardized regression coefficients (β), 95% confidence intervals (95% CI), and p-values with a significance level of p < 0.05. In linear models, the β coefficient represents the average change in the WHOQOL-BREF score for each additional natural tooth. All analyses were performed using SPSS version 23 and were presented in tables prepared using Microsoft Office 2019.

## RESULTS

The data from the present study are available and can be requested from the corresponding author.

A total of 95 participants were included from the communities of Imacita (n=54) and Yamayakat (n=41). The mean age was 74.17 ± 3.21 years in Imacita and 74.81 ± 3.39 years in Yamayakat. The mean number of teeth present was 10 ± 7.57 in Imacita and 8.07 ± 8.28 in the Yamayakat community (p=0.24). The proportion of males was 59.26% in Imacita and 65.85% in Yamayakat, with no significant differences observed (p=0.51). Family type showed a similar distribution, with extended families accounting for 48.15% in Imacita and 48.78% in Yamayakat, without significant differences (p=0.17). Finally, regarding the variable of natural teeth present, complete edentulism was observed in 25.93% of participants in Imacita and 43.90% in Yamayakat, showing a non-significant value (p=0.07) (Table 1).

**Table 1:** Sociodemographic and clinical characteristics of the study population according to community of residence

Variables	Imacita n=54 (100%)	Yamayakat n=41 (100%)	p
**Age, mean ± SD**	74.17 ± 3.21	74.81 ± 3.39	0.35
Natural teeth present, mean ± SD	10 ± 7.57	8.07 ± 8.28	0.24
Male, n (%)	32 (59.26)	27 (65.85)	0.51
**Type of health insurance, n (%)**			
Public	38 (70.37)	34 (82.93)	0.16
Private	16 (29.63)	07 (17.07)	
**Family type, n (%)**			
Extended	26 (48.15)	20 (48.78)	0.95
One-person	28 (51.85)	21 (51.22)	
**Educational level, n (%)**			
Illiterate	11(20.37)	13 (31.71)	0.17
Incomplete primary education	05 (9.26)	03 (7.32)	
Complete primary education	16 (29.63)	11 (26.83)	
Incomplete secondary education	06 (11.11)	0 (0)	
Complete secondary education	12 (22.22)	13 (31.71)	
Higher education	04 (7.41)	01 (2.44)	
**Edentulism, n (%)**			
Completely edentulous	14 (25.93)	18 (43.90)	0.07
Dentate	40 (74.07)	23 (56.10)	
Comparisons between groups were performed using Student’s t-test and the chi-squared test. p<0.05 indicates statistical significance.			

In the bivariate model, the number of natural teeth present was statistically significantly associated with higher quality of life across all WHOQOL-BREF domains (0–100 scale). The strongest associations were observed with satisfaction with general health (β = 1.84 points per tooth; 95% CI: 1.47–2.20; p<0.001) and perceived quality of life (β = 1.62 points per tooth; 95% CI: 1.31–1.92; p<0.001). Significant associations of smaller magnitude were also observed for the physical domain (β = 1.13 points; 95% CI: 0.89–1.36; p<0.001), psychological domain (β = 0.51 points; 95% CI: 0.28–0.75; p<0.001), social domain (β = 0.71 points; 95% CI: 0.36–1.05; p<0.001), and environmental domain (β = 0.47 points; 95% CI: 0.30–0.65; p<0.001) (Table 2).

**Table 2:** Association between the number of natural teeth present and quality-of-life domains using multiple linear regression adjusted for age, sex, and community of residence.

Variable	Bivariate model			Adjusted model		
	F	β (IC 95%)	p	F	β (IC 95%)	p
Perceived overall quality of life	111.84 R² = 0.55 R²Adj = 0.54	1.62 (1.31 – 1.92)	< 0.001*	27.37 R² = 0.55 R²Adj = 0.53	1.54 (1.14 – 1.95)	< 0.001*
Satisfaction with general health	100.53 R² = 0.52 R²Adj = 0.51	1.84 (1.47 – 2.20)	< 0.001*	24.41 R² = 0.52 R²Adj = 0.50	1.85 (1.37 – 2.34)	< 0.001*
Physical domain	92.66 R² = 0.50 R²Adj = 0.49	1.13 (0.89 – 1.36)	< 0.001*	29.28 R²= 0.57 R²Adj = 0.55	0.81 (0.51 – 1.10)	< 0.001*
Psychological domain	18.56 R² = 0.17 R²Adj = 0.16	0.51 (0.28 – 0.75)	< 0.001*	13.30 R² = 0.37 R²Adj = 0.34	0.05 (-0.22 – 0.33)	0.71
Social domain	16.25 R2 = 0.15 R²Adj = 0.14	0.71 (0.36 – 1.05)	< 0.001*	4.35 R² = 0.16 R²Adj = 0.13	0.72 (0.26 – 1.18)	0.002*
Environmental domain	27.36 R² = 0.23 R²Adj = 0.22	0.47 (0.30 – 0.65)	< 0.001*	12.83 R² = 0.36 R²Adj = 0.34	0.17 (-0.05 – 0.38)	0.13
Bivariate model: Quality-of-life domain and number of functional teeth. Adjusted model: Quality-of-life domain and number of natural teeth present, adjusted for age, sex, and community of residence. β: Regression coefficient. 95% CI: Confidence interval for β. F: Overall model test. R²: Coefficient of determination. R^2^Adj: Adjusted coefficient of determination. * p-value < 0.05 for the β coefficient.						

In the model adjusted for age, sex, and community of residence, the associations remained significant for four domains, although with a slight reduction in magnitude (approximately 5%). The most robust associations were observed for satisfaction with general health (β = 1.85 points; 95% CI: 1.37–2.34; p<0.001) and perceived quality of life (β = 1.54 points; 95% CI: 1.14–1.95; p<0.001), followed by the physical domain (β = 0.81 points; 95% CI: 0.51–1.10; p<0.001) and the social domain (β = 0.72 points; 95% CI: 0.26–1.18; p=0.002). This stability suggests a robust relationship between number of teeth and quality of life, not confounded by demographic variables. In contrast, the associations with the psychological (β = 0.05 points; 95% CI: −0.22–0.33; p=0.71) and environmental (β = 0.17 points; 95% CI: −0.05–0.38; p=0.13) domains were not statistically significant (Table 2).

## DISCUSSION

The study observed a significant association in the bivariate model between the number of natural teeth and perceived quality of life, satisfaction with general health, and the physical, psychological, social, and environmental domains. Likewise, after adjustment for age, sex, and community of residence, the association remained significant for quality of life, satisfaction with general health, and the physical and social domains, whereas no association was found for the psychological and environmental domains.

A higher frequency of older adult males was observed in both communities included in the study. In the district of Imaza, where both study communities are located, males account for 49.4% of the population,^28^ while nearly 100% of the population has at least one oral disease.^2^ Previous studies conducted in Indigenous communities have reported that women experience embarrassment regarding dental treatment because they perceive it as lacking privacy,^11^ leading to fear of social judgment and reduced utilization of dental services.^11,40^ In contrast, the higher frequency of older adult males attending dental clinics is largely attributable to treatment-seeking rather than preventive care,^11^ resulting in a greater proportion of male dental visits in populations with a higher burden of oral health problems.^40^

Previous studies have shown that older adults with a lower number of natural teeth tend to prefer soft and sugary foods,^49,52^ which may lead to metabolic dysregulation, affecting mood and perceived energy levels,^52^ and directly impacting self-rated general health.^49^ Other studies conducted in Brazil have reported that living in Indigenous community contexts is associated with cultural differences and inequalities in access to health services,^14,45^ which increases the likelihood of conditions such as pain while chewing and difficulties in speaking,^3^ affecting satisfaction with general health,^4^ and the quality of life of older adults.^7,50^ Consistent with these findings, the present study identified that each additional tooth was associated with an increase of 1.84 points in the domain of satisfaction with general health and 1.62 points in perceived quality of life among older adults; these associations remained consistent regardless of adjustment for age, sex, and community of residence.

The study found that the presence of one additional natural tooth increases the physical domain score of older adults by 1.13 points; this association decreases to 0.81 points after adjustment for age, sex, and community of residence. Although dietary or nutritional variables were not assessed in this study, this association between the number of teeth and physical capacity could be influenced by dietary patterns. Previous studies conducted in China and Chile have reported a similar association among older adults living in Indigenous and rural communities, noting that having more than 20 natural teeth enables the consumption of a varied diet,^5,49^ thereby providing the body with protein, calcium, and vitamins,^49^ which supports the maintenance of muscle mass and physical function, thereby helping to prevent frailty,^4,33^ and reduce the risk of falls.^33^ In this context, evidence suggests that the preservation of natural teeth and masticatory function may act as protective factors for maintaining physical capacity and reducing functional decline during aging,^25,56^ even among older adults living in rural and more vulnerable settings.^25^

Studies conducted in India and Brazil have reported that the number of natural teeth is associated with various social and psychological factors.^50,53^ Recent evidence has shown that individuals living in vulnerable communities are more likely to experience discomfort when eating in front of others and embarrassment when speaking,^37,50^ resulting in reduced social participation and hindering sustained interaction with other members of the community.^39^ Furthermore, tooth loss affects individuals’ self-image, leading to nervousness and embarrassment when smiling.^39^ Both of these circumstances may contribute to the development of depressive symptoms, as they negatively affect psychosocial well-being.^31^ However, many studies have shown that the association between tooth loss and psychological health is linked to other relevant sociodemographic factors,^9^ such as age, sex, income level, chronic diseases, and facial appearance,^9,23^ so that its association with tooth loss disappears after adjustment for these variables.^9,10^ These findings may explain the attenuation observed in the present study, in which the association between number of teeth and the psychological domain was no longer present after adjustment for age, sex, and community of residence. In contrast, it was found that each additional tooth increased the social domain score by 0.71 points, and this association remained statistically significant after adjustment.

In environments with lower socioeconomic status and poorer basic services and accessibility, populations tend to present higher rates of tooth loss.^47,54^ However, previous studies have found that the association between the environmental context and tooth loss is strongly linked to demographic and territorial factors of the population;^14,54^ consequently, statistical significance disappears after adjustment for these variables. These findings are consistent with those of the present study, in which it was determined that the number of teeth and the environmental domain were significantly associated, but this association was lost after adjustment for age, sex, and community of residence.

In summary, the study findings show that the number of natural teeth is associated with certain dimensions of quality of life among older adults in Indigenous communities. Furthermore, to compare the findings with the Minimal Clinically Important Difference (MCID), while recognizing that these thresholds require specific validation in Indigenous populations, values reported for the WHOQOL-BREF instrument, estimated at 8 to 10 points on the 0–100 scale, were used as a reference.^12^ Accordingly, the number of natural teeth required to achieve a clinically meaningful difference was calculated. Regarding the physical domain, 12.3 natural teeth are required to achieve clinically significant differences, whereas 13.8 natural teeth are needed in the social domain to reach this difference. In contrast, the global indicators demonstrated statistically significantly greater sensitivity, with an average of 5.4 natural teeth for health satisfaction and 6.5 natural teeth for overall quality of life. Within this context, the need for interventions aimed at preserving natural dentition in vulnerable, hard-to-reach communities is highlighted in order to maintain physical functioning, social participation, and overall well-being.

Despite the strength of these findings, this study has several limitations that should be considered. First, the cross-sectional design does not allow causal relationships to be established and is limited to demonstrating associations between the number of natural teeth and quality of life. Second, although the sample was representative based on the total population of older adults in the communities, it remained relatively small, which may limit the generalizability of the findings to other populations. Third, clinical variables such as the presence of chronic diseases and the types of medications used daily were not included. This was because Indigenous communities generally do not have medical diagnoses of chronic diseases or routine medication monitoring, as they prioritize urgent healthcare services or treatments that may have been discontinued. However, the absence of these data represents a limitation because these factors may act as confounding variables, potentially influencing the association between the number of natural teeth and quality of life. Fourth, the limited information available regarding the underlying causes of tooth loss, due to the lack of records in hard-to-reach communities, prevented an analysis stratified by etiology, making it difficult to better understand the specific mechanisms underlying the association observed in this study. Finally, the recruitment strategy may have introduced selection bias, as participants recruited through active outreach for dental care were likely to have greater oral health treatment needs compared with the general population, which could have resulted in an overestimation of the observed associations. Despite these limitations, the findings remain valid within the context of the study and provide valuable evidence for future longitudinal research in vulnerable Indigenous populations.

## CONCLUSION

The findings of the present study demonstrate that the number of natural teeth is associated with the quality of life of older adults in indigenous communities of the Peruvian Amazon, independent of age, sex, and community of residence, specifically in the domains of satisfaction with general health, perceived overall quality of life, physical domain, and social domain. In contrast, the psychological and environmental domains lost their association after adjustment for other variables, suggesting that the relationship between these domains and tooth loss is mediated by broader contextual factors. Likewise, the prevalence of edentulism did not show statistically significant differences between the two communities. Based on these findings, the preservation of natural teeth may constitute a protective factor for well-being in vulnerable indigenous populations; therefore, preventive interventions with intercultural approaches are recommended, adapted to the geographic and linguistic barriers characteristic of these settings.

## ACKNOWLEDGEMENT

This research was funded by the Vice-Rectorate of Research of the National University Toribio Rodríguez de Mendoza, Amazonas.
